# How Many Tiers Do We Need? Type I Errors and Power in Multiple Baseline Designs

**DOI:** 10.1007/s40614-020-00263-x

**Published:** 2020-07-29

**Authors:** Marc J. Lanovaz, Stéphanie Turgeon

**Affiliations:** 1 École de psychoéducation, Université de Montréal, C.P. 6128, succursale Centre-Ville, Montreal, QC H3C 3J7 Canada; 2 Centre de recherche de l’Institut universitaire en santé mentale de Montréal, Montreal, QC Canada

**Keywords:** Error rate, Multiple baseline design, Power, Single-case design, Visual analysis

## Abstract

Design quality guidelines typically recommend that multiple baseline designs include at least three demonstrations of effects. Despite its widespread adoption, this recommendation does not appear grounded in empirical evidence. The main purpose of our study was to address this issue by assessing Type I error rate and power in multiple baseline designs. First, we generated 10,000 multiple baseline graphs, applied the dual-criteria method to each tier, and computed Type I error rate and power for different number of tiers showing a clear change. Second, two raters categorized the tiers for 300 multiple baseline graphs to replicate our analyses using visual inspection. When multiple baseline designs had at least three tiers and two or more of these tiers showed a clear change, the Type I error rate remained adequate (< .05) while power also reached acceptable levels (> .80). In contrast, requiring all tiers to show a clear change resulted in overly stringent conclusions (i.e., unacceptably low power). Therefore, our results suggest that researchers and practitioners should carefully consider limitations in power when requiring all tiers of a multiple baseline design to show a clear change in their analyses.

In behavior analysis, researchers and practitioners typically use single-case designs such as the reversal design, the alternating-treatment design, and the multiple baseline design to demonstrate experimental control (Gast & Ledford, 2018; Horner et al., 2005; Kratochwill et al., 2010). Among these designs, researchers have found that multiple baseline designs were the most frequently used (Coon & Rapp, 2018; Shadish & Sullivan, 2011; Smith, 2012). In contrast with other single-case designs, the multiple baseline design does not require the withdrawal of the treatment or the establishment of a criterion to be gradually changed, which may explain its predominant use in single-case research (Baer, Wolf, & Risley, 1968; Kratochwill & Levin, 2014).

The multiple baseline design involves the sequential introduction of an independent variable across behaviors, contexts, or participants (see Fig. 1 for two examples of multiple baseline graphs). When analyzing multiple baseline graphs, experimenters depict each behavior, context, or participant with a different AB comparison. We may refer to each of these individual AB comparisons as tiers. For example, the left graph of Fig. 1 contains three different tiers. Each tier must remain functionally independent; that is, the introduction of the independent variable in one tier should not be expected to produce changes in another tier (i.e., behavior, context, or participant). When the purpose of the study is to demonstrate experimental control, the experimenter should only introduce the independent variable in a tier when the previous tier (i.e., preceding AB comparison) shows a clear change.

**Fig. 1:**
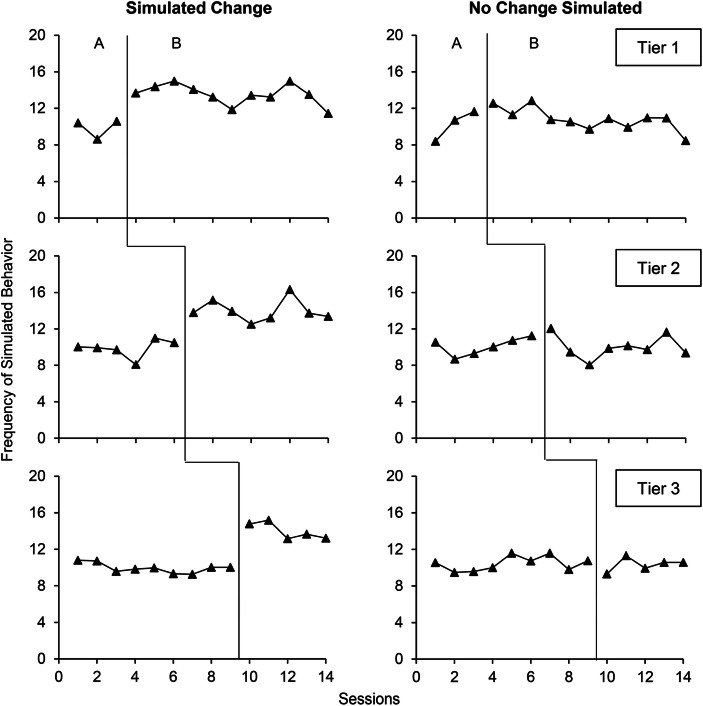
Multiple Baseline Graphs wherein the Observers and the Dual-Criteria Method Always Agreed on the Presence or Absence of a Clear Change

Through the initiative of the What Works Clearinghouse (WWC), Kratochwill et al. (2010) developed highly cited guidelines for evidence-based single-case research, which include specific design criteria for multiple baseline designs. For multiple baseline designs to meet the criteria without reservation, the introduction of the independent variable must be staggered across tiers, the design must contain a minimum of three AB tiers, and each phase must have at least five data points. For the results of a three-tier multiple baseline design to qualify as strong evidence of a functional relationship between the independent and the dependent variables, all three temporally independent replications must demonstrate an effect. This criterion is not unique to the WWC guidelines: Other standards to evaluate the quality of single-case designs recommend three demonstrations, too (e.g., Tate et al., 2013). In a recent textbook, Gast, Lloyd, and Ledford (2018) also suggest that researchers and practitioners should aim for three or four demonstrations of effects when using a multiple baseline design.

Despite its widespread adoption, the current recommendation of requiring three demonstrations of effects does not appear grounded in empirical evidence. One concern is that this recommendation may be overly stringent. Assuming that the analysis of AB designs had an average power of .80, we would statistically expect the three tiers of multiple baseline designs to agree only 51.2% of the time (i.e., .80^3^) in the presence of a true effect. Thus, the current recommendation would lead to erroneous conclusions in a large proportion of cases. The main purpose of our study was to address this issue by assessing Type I error rate (false positives) and power in multiple baseline designs with varying numbers of tiers.

## Study 1: Error Rate and Power with a Structured Visual Aid

The most studied structured visual aids in single-case research are the dual-criteria and conservative dual-criteria methods (Fisher, Kelley, & Lomas, 2003). Several researchers have shown that these methods adequately control for errors of analyses in single-case AB designs (Falligant, McNulty, Hausman, & Rooker, 2020; Lanovaz, Huxley, & Dufour, 2017; Manolov & Vannest, 2020). To apply the dual-criteria method, the researcher or practitioner must first draw the baseline (i.e., Phase A) data mean and trend lines. Both lines are then extended into the intervention phase of the comparison (i.e., Phase B). To assess the reliability of an intervention effect, the researcher must compare the number of data points in the intervention phase that fall above or below both lines to a threshold quantity. The conservative dual-criteria method is similar; however, it requires raising or lowering the mean and trend lines by 0.25 standard deviations. Despite being specifically recommended for multiple baseline designs (Fisher et al., 2003; Swoboda, Kratochwill, & Levin, 2010), we found no studies examining its error rate when applied to this type of design. In our first study, we applied the dual-criteria method to individual tiers, and then examined the Type I error rate and power based on different numbers of tiers in multiple baseline graphs.

### Dataset

The first author programmed R code to generate the multiple baseline graphs in the dataset by adapting code developed by Lanovaz et al. (2020). Each multiple baseline graph had five tiers with 20 points each. To remain consistent with the results of prior research, Phase A never contained fewer than three points whereas Phase B never fewer than five points (Falligant et al., 2020; Lanovaz, Giannakakos, & Destras, 2017). Moreover, each tier within a multiple baseline graph had three more baseline points than the previous tiers while maintaining an equal number of total points (i.e., 20 points). Thus, the number of points for Phase A and Phase B were respectively, 3 and 17 for the first tier, 6 and 14 for the second tier, 9 and 11 for the third tier, 12 and 8 for the fourth tier, and 15 and 5 for the final tier.

To generate the values of the multiple baseline graphs, R code first used the following equation for each tier:

$$ {x}_t={ax}_{t-1}+{\varepsilon}_t $$

where *x* was a univariate times series, *t* an index of time (i.e., 1 to 20), *a* the autocorrelation value^1^ and ε a normally distributed error term with a mean of 0 and a standard deviation of 1. The autocorrelation term had a value that varied randomly according to a normal distribution with a mean of .20 and a standard deviation of .15 (see Lanovaz et al., 2020; Shadish & Sullivan, 2011). The autocorrelation remained consistent across tiers within the same multiple baseline graph. Then, we transformed the univariate time series using this second equation:

$$ {y}_t={x}_t+c+ SMD $$

where *y*_*t*_ was the value of a data point on session *t*, *c* was a constant of 10 to avoid negative values, and SMD the standardized mean difference. The R code generated graphs in pairs that each had the same autocorrelation value. In the first graph of the pair, the SMD remained at zero for all data points. In the second graph of the pair, we set the SMD at zero for points in Phase A whereas we added a SMD value to points in Phase B. This latter SMD value varied randomly according to a normal distribution with a mean of 3 and a standard deviation of 1. The value remained the same across tiers within the same multiple baseline graph but varied across graphs.

We used the first graph (with no SMD) of each pair to measure Type I error rate and the second graph (with SMD added to Phase B) to assess power. The code generated 10,000 graphs in total: 5,000 simulated no effect and the other 5,000 simulated an effect. The raw data and code used for the analyses are freely available on the Open Science Framework for replications and extensions (https://osf.io/5ua3v/).

### Analyses

Given that our concern involved power and that the dual-criteria method is more powerful than the conservative dual-criteria method (Fisher et al., 2003), our study only applied the dual-criteria method. The analyses first involved applying the dual-criteria method to all tiers for each graph (i.e., 50,000 tiers in total). The function recorded a score of 1 if the graph showed a clear change according to the dual-criteria method and a 0 when there was no clear change.

The next step entailed computing Type I error rate and power depending on the number of tiers that showed a clear change for one to five tiers. In single-case research, a Type I error occurs when an analysis method (e.g., dual-criteria method, visual inspection) concludes that the independent variable produced a change in behavior when there was no true change in behavior. In this step, our R code computed Type I error rate by dividing the number of graphs with no simulated effect that had a specific number of tiers showing a clear change (according to the dual-criteria analysis) by the total number of graphs with no simulated effect.

In contrast, power represents the proportion of tiers showing a true clear change that were correctly identified by the analysis method. Calculating power involved dividing the number of graphs with a simulated effect that had a specific number of tiers showing a clear change (as indicated by the dual-criteria analysis) by the total number of graphs with a simulated effect. We repeated these analyses for all combinations of tiers. For example, when analyzing graphs with three tiers, our code extracted all possible combinations of three tiers for each graph (out of five) and then examined the number of tiers showing a clear change. Next, our analysis involved measuring Type I error rate and power when we required one or more of three tiers to show a clear change, two or more of three tiers to show a clear change, and three of three tiers to show a clear change.

### Results and Discussion

Table 1 presents the Type I error rate and the power based on the number tiers that needed to show a clear change. Analyzing tiers individually indicated that the dual-criteria method produced a Type I error rate of .08 and a power of .80. As expected, Type I error rate and power decreased when the number of tiers showing a clear change increased. The only analyses that produced both adequate Type I error rate (> .05) and power (< .80) are those with at least three tiers in total. To adequately control for errors, the multiple baseline needed to include at least two tiers with a clear change for graphs with three tiers, and at least two or three tiers with a clear change for graphs with four tiers or five tiers. It should be noted that requiring three of three tiers to demonstrate a clear change, which is often the case in single-case research, would lead researchers and practitioners in drawing incorrect conclusions in more than 40% of cases showing a true effect (assuming that the distribution of parameters in nonsimulated graphs is similar to the one simulated in the current study). Because this result runs counter to a previously established recommendation and three-tier multiple baseline designs are common in research, we conducted a more in-depth analysis of designs with three tiers using visual inspection.

**Table 1.** Type I Error Rate and Power Based on the Number of Tiers that Show a Clear Change

Total Number of Tiers	Number of Tiers with Clear Change				
	At Least 1	At Least 2	At Least 3	At Least 4	5
1					
Type I Error Rate	.077				
Power	.804				
2					
Type I Error Rate	.149	.006			
Power	.951	.658			
3					
Type I Error Rate	.215	.017	.001		
Power	.982	.888	.542		
4					
Type I Error Rate	.276	.031	.002	<.001	
Power	.992	.953	.823	.448	
5					
Type I Error Rate	.333	.049	.005	<.001	<.001
Power	.996	.975	.919	.760	.370

*Note.* The highlighted numbers identify combinations of tiers that simultaneously achieved a Type I error rate lower than .05 and a power higher than .80

## Study 2: Error Rate and Power with Visual Inspection

For single-case research, decision on the effect of the independent variable primarily relies on visual inspection of graphs, which is also recommended by the WWC guidelines (Kratochwill et al., 2010, 2013). Although visual inspection remains a subjective practice (Manolov & Vannest, 2020; Ninci, Vannest, Willson, & Zhang, 2015; Wolfe, Seaman, Drasgow, & Sherlock, 2018), power of visual inspection is largely undocumented. As such, one may argue that visual inspection could be more powerful than the dual-criteria method and circumvent the issues observed in the first study. We thus replicated and extended our first study by having expert raters visually inspect each tier.

### Dataset

To reduce the number of tiers and graphs to visually inspect, our second study involved 300 new graphs with three tiers containing 14 points each. The other parameters and the data generation procedures remained the same as in the first study.

### Visual Inspection

We hired two independent raters blind to the purpose of the current study to conduct the visual inspection. Each rater was a college-level professor in a course sequence verified by the Association for Behavior Analysis International® and a Board Certified Behavior Analyst-Doctoral® (BCBA-D). The raters inspected each tier individually (900 in total) and responded to the following question, “Would the change observed from Phase A to Phase B be indicative of functional control showing an increase in behavior if similar patterns were replicated across two additional multiple baseline tiers?” The raters recorded a positive response (i.e., 1) when the tier showed a clear change and a negative response (i.e., 0) when the tier showed no clear change. To prevent bias, each tier was presented individually on a page and the order of presentation of tiers was randomized. Therefore, the raters remained unaware of the multiple baseline graph to which each tier belonged and of the results of the other rater.

### Analyses

First, the R code repeated the analyses described in the first study with the data from the second study using the dual-criteria analysis, the inspection from the first rater, and the inspection from the second rater. As power remained an issue following this analysis, we examined the effects of autocorrelation and effect size on power. For autocorrelation, our functions split the graphs showing a simulated effect in two based on the mean of our equation (i.e., autocorrelation < .2 vs. autocorrelation > .2) and then computed power for each half separately. The R code repeated the same procedure for the mean effect size in our equation (i.e., *SMD* < 3 vs. *SMD* > 3).

### Results and Discussion

When considering each tier individually, the Type I error rate was .09 for the dual-criteria method, .05 for the first rater, and .02 for the second rater. Power was .74, .89, and .69 for the dual-criteria method, the first observer, and the second observer, respectively. Agreement between the two raters was .87. Table 2 presents the Type I error rate and power based on the number tiers that needed to show a clear change for each analysis. The Type I error rate remained near or below .01 for all analyses, indicating that it was not a concern when analyzing multiple baseline graphs. On the other hand, power varied considerably across analyses. The dual-criteria method produced adequate power only when at least two of three tiers showed a clear change. Rater 1 achieved adequate power for two of two tiers and at least two of three tiers, and nearly attained a power of .80 for three of three tiers. In contrast, the analyses of the second rater never produced adequate power. Similar to the first rater, the power of the second rater was also highest for at least two of three tiers showing a clear change.

**Table 2.** Type I Error Rate and Power Based on the Dual-Criteria Method and the Visual Analysis of Two Raters

	Number of Tiers with Clear Change		
	2 of 2	At Least 2 of 3	3 of 3
Dual-Criteria Method			
Type I Error Rate	.004	.013	0
Power	.587	.813	.473
Rater 1			
Type I Error Rate	.004	.013	0
Power	.831	.907	.793
Rater 2			
Type I Error Rate	0	0	0
Power	.518	.753	.400

*Note.* The highlighted numbers identify combinations of tiers that simultaneously achieved a Type I error rate lower than .05 and a power higher than .80

**Fig. 2:**
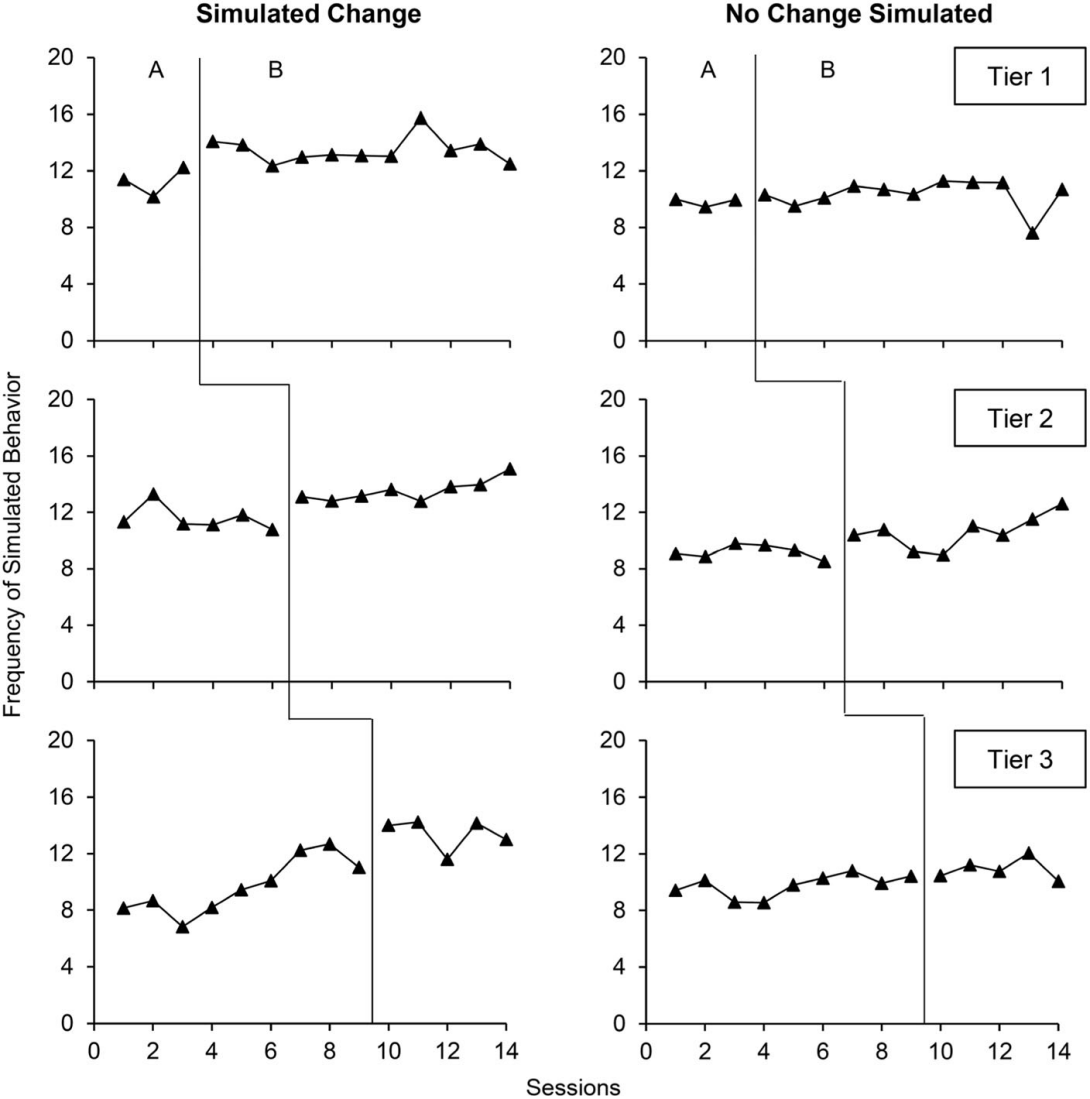
Multiple Baseline Graphs wherein the Observers and the Dual-Criteria Method Disagreed on the Presence or Absence of a Clear Change

Figure 1 shows an example of two multiple baseline graphs wherein the two raters and the dual-criteria method all agreed on the presence or absence of a clear change. For the left graph, the analyses agreed that all tiers showed a clear change. For the right graph, the opposite was true: each analysis concluded that all tiers showed no clear change. Figure 2 shows two examples of multiple baseline graphs wherein the raters and dual-criteria method disagreed. The left graph showed a simulated change in all tiers. Only the first rater detected a clear change in all tiers. The dual-criteria method failed to detect a clear change in the first and second tiers whereas the second rater only detected a clear change in the first tier. The right graph showed no simulated change in all tiers. Despite this lack of simulated effect, the dual-criteria method detected a change in the first two tiers and the first rater in the last two tiers. In this example, the second rater correctly concluded that there was no clear change in all tiers. These latter results show that different raters may perform inconsistently on graphs with different distributions (i.e., the first rater scored the left graph correctly whereas the second rater performed adequately on the right graph).

Table 3 shows the power of the analyses across different values of autocorrelation and SMD. Higher autocorrelation values led to marginally lower power. In contrast, high values of SMD produced considerably higher power. Only the first rater produced acceptable power for both small and large autocorrelation and SMD values but only when two of two tiers or at least two of three tiers showed a clear change.

**Table 3.** Power across Smaller and Larger Values of Autocorrelation and Standardized Mean Difference

	Number of Tiers with Clear Change		
	2 of 2	At Least 2 of 3	3 of 3
Dual-Criteria Method			
*a* < 0.20	.605	.831	.492
*a* > 0.20	.573	.800	.459
*SMD* < 3	.406	.679	.269
*SMD* > 3	.782	.958	.694
Rater 1			
*a* < 0.20	.867	.969	.815
*a* > 0.20	.804	.859	.776
*SMD* < 3	.675	.821	.603
*SMD* > 3	1	1	1
Rater 2			
*a* < 0.20	.585	.831	.462
*a* > 0.20	.467	.694	.353
*SMD* < 3	.295	.551	.167
*SMD* > 3	.759	.972	.653

*Note.* The highlighted numbers identify combinations of tiers where power was at least .80 regardless of the parameter values. *a* = autocorrelation, *SMD* = standardized mean difference

## General Discussion

Overall, our results suggest that requiring all tiers to show a clear change in multiple baseline designs may be an overly stringent practice. When a design has at least three tiers and two or more of these tiers show a clear change, the Type I error rate remained adequately controlled while power also reached acceptable levels. This result seems inconsistent with the WWC recommendation requiring at least three demonstrations of effects within a multiple baseline to consider an experiment as adequate without reservation. Even when a design only contains three tiers, requiring at least two tiers to be significant leads to few Type I errors. The advantage of this approach is a considerable increase in power. Requiring all tiers to show a clear change may increase the burden of proof on behavior analysts and lead to the rejection of smaller or more variable (true) effects. To understand human behavior, we cannot discard true effects only because they are small or display high variability, which is what the current recommendation may be doing.

Taken together, the results of the two raters support concerns involving the reliability of visual inspection (DeProspero & Cohen, 1979; Fisher et al., 2003; Ninci et al., 2015). Given that both raters were college-level professors with the same level of certification, we would have expected them to produce similar conclusions based on the data. Although agreement was .87 on individual tiers and both raters adequately controlled for Type I error rate, the analysis of power shows how small differences in visual inspection between two raters may be amplified to produce different conclusions regarding the effect of an intervention. For example, the first rater had power near or above .80 for all combinations of tiers tested whereas the second rater never achieved this criterion. It is important to note that the first rater clearly demonstrates that it is possible for visual inspection to outperform the dual-criteria method. Given that the two raters had similar credentials, our results indicate that training in visual inspection needs to be further improved and systematized (e.g., Retzlaff, Phillips, Fisher, Hardee, & Fuhrman, 2020; Stewart, Carr, Brandt, & McHenry, 2007).

Our study may have produced different results if the raters had been allowed to analyze the multiple baseline graphs as a whole (rather than individual tiers) or use analysis methods that consider all tiers simultaneously (Bouwmeester & Jongerling, 2020). If two of three tiers show a clear change and the final tier is ambiguous, some practitioners and researchers may overlook this last tier. For example, Wolfe, Seaman, and Drasgow (2016) found that 35% of their raters considered that two of three tiers with clear changes was sufficient to show a functional relation in a study on the visual analysis of multiple baseline graphs. Moreover, researchers should manipulate other variables such trend and variability (e.g., DeProspero & Cohen, 1979; Kahng et al., 2010) to examine their impact in the future because these dimensions may interact with others to influence responding.

The study has some additional limitations that should be noted. First, we arbitrarily set the initial the SMD value at a mean of 3, which is considered as relatively small in single-case research (Rogers & Graham, 2008; Lanovaz et al., 2020). Because smaller changes typically have lower power, replicating our study with larger SMD values may produce different outcomes. Second, our study limited its analysis to the dual-criteria method and visual inspection. In the future, researchers may also explore other methods of data analyses to examine whether the results remain consistent (e.g., Krueger, Rapp, Ott, Lood, & Novotny, 2013; Manolov & Vannest, 2020). Third, our analyses only involved simulated data as it is impossible to measure power on nonsimulated data without delving into circular reasoning. Nevertheless, researchers could examine Type I error rate on nonsimulated data in the future (Falligant et al., 2020; Lanovaz et al., 2017). Finally, the distribution of our simulation data was limited by our choice of starting parameters (e.g., autocorrelation values, SMD), which may not perfectly represent the distribution of data in nonsimulated graphs. Until our results are replicated by an independent research team, we do not recommend that practitioners and researchers adopt a new criterion involving the analysis of multiple baseline design (e.g., requiring at least two of three tiers showing a clear change). That said, researchers and practitioners should carefully consider limitations in power when requiring all tiers of a multiple baseline design to show a clear change in their analyses.

## Footnotes

1 Autocorrelation in single-case graphs refers to the observation that the frequency or duration of a behavior during a session is often correlated with the frequency or duration of this same behavior in prior sessions.
